# Associations of fish oil supplementation with incident dementia: Evidence from the UK Biobank cohort study

**DOI:** 10.3389/fnins.2022.910977

**Published:** 2022-09-07

**Authors:** Yan Huang, Yajuan Deng, Peizhen Zhang, Jiayang Lin, Dan Guo, Linjie Yang, Deying Liu, Bingyan Xu, Chensihan Huang, Huijie Zhang

**Affiliations:** ^1^Department of Endocrinology and Metabolism, Nanfang Hospital, Southern Medical University, Guangzhou, China; ^2^Guangdong Provincial Key Laboratory of Shock and Microcirculation, Guangzhou, China; ^3^Department of Food Safety and Health Research Center, School of Public Health, Southern Medical University, Guangzhou, Guangdong, China

**Keywords:** fish oil supplementation, polyunsaturated fatty acid, dementia, vascular dementia, frontotemporal dementia, Alzheimer’s disease, UK Biobank cohort

## Abstract

**Background:**

Although numerous studies have investigated the association of dietary intake of omega-3 fatty acids with cognitive function and the risks of dementia, the relationship between fish oil supplementation and incident dementia in a large population-based cohort study has not yet well studied.

**Materials and methods:**

A total of 211,094 community-dwelling older persons over 60 years from the UK Biobank cohorts enrolled between 2006 and 2010 that reported regularly taking fish oil and had no dementia at baseline, was included in the present study. All participants completed an electronic questionnaire regarding habitual use of supplements including fish oil.

**Results:**

Overall, 83,283 (39.5%) participants reported regularly taking fish oil at baseline. Of 211,094 participants with the median age was 64.1 years, 5,274 participants developed dementia events during a median follow-up of 11.7 years, with 3,290 individuals derived from fish oil non-users. In the multivariable adjusted models, the adjusted hazard ratios (HRs) associated with fish oil supplementation for all-cause dementia, vascular dementia, frontotemporal dementia, and other dementia were 0.91 [CI = 0.84–0.97], 0.83 [CI = 0.71–0.97], 0.43 [CI = 0.26–0.72], 0.90 [CI = 0.82–0.98], respectively (all *P* < 0.05). However, no significant association between fish oil supplementation and Alzheimer’s disease was found (HR = 1.00 [CI = 0.89–1.12], *P* = 0.977). In the subgroup analyses, the associations between use of fish oil and the risk of all-cause dementia (*P* for interaction = 0.007) and vascular dementia were stronger among men (*P* for interaction = 0.026).

**Conclusion:**

Among older adults, regular fish oil supplementation was significantly associated with a lower risks of incident all-cause dementia, as well as vascular dementia, frontotemporal dementia and other dementia but not Alzheimer’s disease. These findings support that habitual use of fish oils may be beneficial for the prevention of dementia in clinical practice.

## Introduction

Dementia has been emerged as a major global public health issue because of worldwide aging population. It is estimated that approximately 50 million people worldwide have dementia such as Alzheimer’s disease, vascular dementia and frontotemporal dementia, and its prevalence is expected to almost triple by 2050 (Scheltens et al., 2016). Although no licensed drugs are available to prevent or reverse dementia (Jennings et al., 2020), the proactive management of modifiable risk factors such as dietary and lifestyle factors has been established to prevent dementia (WHO, 2019). Nutrition has raised great interest for dementia prevention, and the long-chain omega-3 polyunsaturated fatty acid mainly provided by dietary fish intake, such as eicosapentaenoic acid (EPA) and docosahexaenoic acid (DHA), is implicated in many aspects of neuroprotective and anti-inflammatory properties that may preserve brain vasculature and collectively contribute to lower neurodegeneration and maintain cognitive functioning during aging (Daiello et al., 2015). Consequently, the use of fish oil supplements is initially recommended to prevent cognitive decline (Lim et al., 2006), and is widespread in aging population worldwide and promising candidates for dementia.

Although there have been marked advances in recent years in our understanding of the role of omega-3 fatty acids on dementia or cognition, clear gaps in knowledge remain, and studies have yielded inconsistent and mixed findings (van de Rest et al., 2009; Gustafson et al., 2020). Observational studies reported that higher levels of plasma omega-3 fatty acids, and higher fish or long-chain omega-3 fatty acids intakes were associated with lower risk of dementia risk (Thomas et al., 2020; Nozaki et al., 2021) or lower cognitive decline (Kalmijn et al., 2004; Nishihira et al., 2016). Furthermore, one small randomized controlled trial (RCT) reported that omega-3 supplementation significantly slowed cognitive decline in at-risk elderly people (Sinn et al., 2012). In contrast, another clinical intervention study of 2,911 patients with stable myocardial infarction reported no effect of n-3 fatty acids supplementation on cognitive decline (Geleijnse et al., 2012). In LipiDiDiet study of 311 patients with prodromal Alzheimer’s disease, the multinutrient combination (contains DHA and EPA) also showed no significant effect on the neuropsychological test battery score primary endpoint during the 24-month intervention (Soininen et al., 2017), whereas intervention benefits increased with 36-months use (Soininen et al., 2021). However, these RCT evidences are more limited and less convincing, with limited sample sizes and cognition or brain volume as main study endpoints (Chiu et al., 2008; Quinn et al., 2010; Arellanes et al., 2020),and few primary preventions study with incident dementia as an outcome measure. Furthermore, insufficient sample sizes might have limited the statistical power of RCTs to detect the clinical effects of fish oil supplementation.

Besides, clinical RCT evidence from fish oil supplements is difficult to generalize to larger and more inclusive population because of the performance of RCTs under ideal and controlled circumstances. Therefore, complementary information regarding the effectiveness of fish oil supplements on dementia prevention is needed to investigate in real life settings of large-scale cohort studies. In the present study, we used a large population-based cohort data from the UK Biobank study to investigate the associations of fish oil supplementation with the risks of incident dementia and its subtypes.

## Materials and methods

### Study design and participants

Between 2006 and 2010, UK Biobank recruited over 500,000 participants from the general population across the United Kingdom (Collins, 2012; Sudlow et al., 2015). Participants attended one of 22 assessment centers across England, Scotland, and Wales where they completed a touchscreen questionnaire and face-to-face interview, took various physical measurements, and reported medical conditions. Data from 502,490 participants were available for this study. Considering that the majority of incident dementia cases occur in older adults, participants aged less than 60 years (*N* = 285,012) were excluded in this analysis. In addition, participants with self-reported prevalent cognitive impairment or dementia at baseline or prevalent dementia diagnosis based on hospital inpatient records (*N* = 166) at baseline, those who subsequently withdrew from the study (*N* = 4), and those with incomplete information on covariates, the use of fish oil and the intake of oily fish (*N* = 6 214) were also excluded. In total, this analysis included 211,094 participants.

### Ascertainment of exposure

The habitual use of fish oil supplements was assessed when participants attended the UK Biobank cohort study. Participants were asked, “Do you regularly take any of the following?” and participants could select their answer from a list of supplements using a touchscreen questionnaire at baseline. From this information, we defined fish oil use as “0 = no” or “1 = yes” in this study.

### Ascertainment of outcomes

The main outcomes of the current study included the incidence of all-cause dementia and specific types of dementia (i.e., Alzheimer’s disease, vascular dementia, frontotemporal dementia, and other dementia). Data on hospital admissions were collected regularly through linkages to Health Episode Statistics, the Patient Episode Database, and the Scottish Morbidity Records, which was a part of UK Biobank dataset. Information on death was obtained from National Health Service (NHS) Digital for participants in England and Wales and from the NHS Central Register (NHSCR), part of the National Records of Scotland, for participants in Scotland. For the analyses of main outcomes, we censored follow-up at 30 November 2020 or the date of death, whichever occurred first. The diagnosis of dementia was ascertained using the International Classification of Diseases (ICD) coding system from hospital records and death register: Alzheimer’s disease codes: F00, F00.0, F00.1, F00.2, F00.9, G30, G30.0, G30.1, G30.8, and G30.9; vascular dementia codes: F01, F01.0, F01.1, F01.2, F01.3, F01.8, F01.9, and I67.3; frontotemporal dementia codes: F02.0, G31.0; other codes for all-cause dementia: A81.0, F02, F02.1, F02.2, F02.3, F02.4, F02.8, F03, F05.1, F10.6, G31.1, and G31.8).

### Ascertainment of covariates

The potential confounding variables includingsociodemographic factors (age, gender, ethnicity, and education), lifestyle habits (smoking status, drinking status), physical measurements [body mass index (BMI)], medications (antihypertensive drug use, Lipid lowering medication, and aspirin use), and mineral and other dietary supplementation (calcium, iron, zinc, or selenium), and medical history (hypertension, diabetes) were obtained from the baseline questionnaire. Education categorized as college or above, high school or equivalent, less than high school and vocational. Prevalent hypertension was defined as an ICD-10 diagnosis, self-reported history of hypertension, the use of antihypertensive medication, a systolic blood pressure of 140 mm Hg or higher, or a diastolic blood pressure of 90 mm Hg or higher at baseline. Prevalent diabetes was defined as an ICD-10 diagnosis, self-reported history of diabetes, the use of antidiabetics medication, glucose ≥7.0 mmol/L or serum glycosylated hemoglobin >48 mmol/mol at baseline. Further details of covariate measurements can be found in the UK Biobank online protocol.^1^

### Statistical analysis

Statistical analyses were performed using statistical analysis system (SAS) 9.4 (SAS Institute Inc., Cary, NC, United States). *P* < 0.05 was considered statistically significant. Baseline characteristics are presented as means (standard deviation, SD) or median (interquartile range) for the continuous variables and number (percentage) for categorical variables. The generalized linear model was used to examine the baseline characteristics of participants among groups.

The Cox proportional hazard models were used to calculate the hazard ratios (HRs) and 95% confidence intervals (CIs) for the associations of habitual fish oil supplementation with outcomes. The multivariable model (Model 1) was adjusted for age and sex; Model 2 was further adjusted for ethnicity, education level, smoking status, alcohol consumption, presence of hypertension, antihypertensive drug use, presence of diabetes, aspirin use, lipid lowering medication, other vitamin or mineral or dietary supplementations, Townsend Deprivation Index, household income, and physical activity. Stratified analyses were performed according to gender (male or female), ethnicity (white or non-white), current smoking status (yes or no), current drinking status (yes or no), prevalent hypertension (yes or no), prevalent diabetes (yes or no), obesity (<30 or ≥30 Kg/m^2^), lipid lowering medication (yes or no). Further stratified analyses were also conducted according to the frequency of oily fish intake (<2 or ≥2 times/week). To investigate whether the associations between habitual fish oil supplementation and outcomes differed by these stratification variables, the potential effect modification was examined using the interaction models.

## Results

### Baseline characteristics

Baseline characteristics of the participants stratified by fish oil supplementation status (non-users vs. users) were presented in Table 1. Totally, 111,583 (52.9%) participants were female, 205,193 (97.2%) were white and 81,444 (38.6%) had college or above education level, with a mean (SD) age of 64.1 (2.9) years. Of 211,094 participants, 83,283 (39.5%) reported regularly take fish oil at baseline. Compared with the fish oil non-users, fish oil users were older, more likely to be female, no current drinkers, current smokers, physically active, and had less BMI (all *P* < 0.001). Furthermore, fish oil users had higher frequency of oily fish intake and had lower prevalence of hypertension and diabetes than non-users (all *P* < 0.001). Likewise, fish oil users were more likely to take aspirin, mineral and other dietary supplementations, and had lower percentage of using antihypertensive drug and lipid lowering medication than the non-users (all *P* < 0.001).

**TABLE 1:** Baseline characteristics of the study participants stratified by fish oil use.

Characteristics	Overall (*N* = 211,094)	Fish oil non-users (*N* = 127,811)	Fish oil users (*N* = 83,283)	*P*-value
Mean age (SD) (*years*)	64.1 (2.9)	64.0 (2.8)	64.3 (2.9)	<0.001
Female (*n*, %)	111,583 (52.9)	65,021 (50.9)	46,562 (55.9)	<0.001
Townsend Deprivation Index	−1.57 (2.95)	−1.46 (3.02)	−1.75 (2.8)	<0.001
Ethnicity (*n*, %)				<0.001
White	205,193 (97.2)	124,061 (97.1)	81,132 (97.4)	
Asian	2,477 (1.2)	1,751 (1.4)	726 (0.9)	
Black	1,534 (0.7)	873 (0.7)	661 (0.8)	
Chinese	297 (0.1)	161 (0.1)	136 (0.2)	
Mixed	611 (0.3)	356 (0.3)	255 (0.3)	
Others	982 (0.5)	609 (0.5)	373 (0.5)	
Education level (*n*, %)				<0.001
College or above	81,444 (38.6)	49,830 (39.0)	31,614 (38.0)	
High school or equivalent	15,930 (7.6)	9,362 (7.3)	6,568 (7.9)	
Less than high school	98,866 (46.8)	59,748 (46.8)	39,118 (47.0)	
Vocational	14,854 (7.0)	8,871 (6.9)	5,983 (7.2)	
Smoking status (*n*, %)				<0.001
Never	105,511 (50.0)	63,250 (49.5)	42,261 (50.7)	
Previous	88,236 (41.8)	52,649 (41.2)	35,587 (42.7)	
Current	17,347 (8.2)	11,912 (9.3)	5,435 (6.5)	
Drinking status (*n*, %)				<0.001
Never	9,960 (4.7)	6,529 (5.1)	3,431 (4.1)	
Previous	7,977 (3.8)	5,218 (4.1)	2,759 (3.3)	
Current	193,157 (91.5)	116,064 (90.8)	77,093 (92.6)	
Household income (£)				
<18,000*	58,550 (33.8)	35,890 (34.1)	22,660 (33.3)	<0.001
18,000−30,999	56,668 (32.7)	33,394 (31.8)	23,274 (34.2)	
31,000−51,999	36,322 (21.0)	21,881 (20.8)	14,441 (21.2)	
52,000−100,000	17,534 (10.1)	11,104 (10.6)	6,430 (9.4)	
>100,000	4,173 (2.4)	2,850 (2.7)	1,323 (1.9)	
Physical activity (*min/week*)				<0.001
<150	81,128 (41.3)	51,922 (43.8)	29,206 (37.5)	
≥150	115,474 (58.7)	66,713 (56.2)	48,761 (62.5)	
Oily fish intake (*times/week*)				<0.001
<2	164,462 (77.9)	102,015 (79.8)	62,447 (75.0)	
≥2	46,632 (22.1)	25,796 (20.2)	20,836 (25.0)	
Mean (SD) Body mass index (*Kg/m*^2^)	27.6 (4.5)	27.8 (4.7)	27.3 (4.4)	<0.001
Hypertension (*n*, %)	161,372 (76.5)	98,061 (76.7)	63,311 (76.0)	<0.001
Diabetes (*n*, %)	19,528 (9.3)	12,984 (10.2)	6,544 (7.9)	<0.001
Antihypertensive drug use (*n*, %)	28,943 (13.7)	17,832 (14.0)	11,111 (13.3)	<0.001
Lipid lowering medication (*n*, %)	58,365 (27.7)	36,276 (28.4)	22,089 (26.5)	<0.001
Aspirin use (*n*, %)	44,298 (21.0)	26,364 (20.6)	17,934 (21.5)	<0.001
Mineral and other dietary supplementation (*n*, %)	99,187 (47.0)	38,920 (30.5)	60,267 (72.4)	<0.001

*£18,000 = €21,489; $23,253. Data are presented as the mean (SD) or median (interquartile range) or numbers (percentages).

### Fish oil supplementation and dementia

Table 2 shows the associations of fish oil supplementation with all-cause dementia and its subtypes. During a median follow-up of 11.7 years, 5,274 participants developed incident all-cause dementia events, with 3,290 (2.6%) derived from fish oil non-users and 1,984 (2.4%) derived from fish oil users. Furthermore, the incident cases of Alzheimer’s disease, vascular dementia, frontotemporal dementia, and other dementia were 1,351 (1.1%), 800 (0.6%), 111 (0.1%), and 2,302 (1.8%) among fish oil non-users, respectively; and the incident cases were 911 (1.1%), 424 (0.5%), 42 (0.1%), and 1,366 (1.6%) among fish oil user, respectively.

**TABLE 2:** Associations of use of fish oil supplements with the risk of dementia.

Outcomes (*n*, %)	Fish oil non-users	Fish oil users	Crude		Model 1		Model 2	
	(*n* = 127,811)	(*n* = 83,283)	HR (95% CI)	*P*-value	HR (95% CI)	*P*-value	HR (95% CI)	*P*-value
All-cause dementia	3,290 (2.6)	1,984 (2.4)	0.91 (0.86–0.96)	<0.001	0.88 (0.83–0.93)	<0.001	0.91 (0.84–0.97)	0.007
Alzheimer’s disease	1,351 (1.1)	911 (1.1)	1.01 (0.93–1.10)	0.762	0.97 (0.90–1.06)	0.527	1.00 (0.89–1.12)	0.977
Vascular dementia	800 (0.6)	424 (0.5)	0.80 (0.71–0.90)	<0.001	0.78 (0.69–0.87)	<0.001	0.83 (0.71–0.97)	0.019
Frontotemporal dementia	112 (0.1)	42 (0.1)	0.57 (0.40–0.81)	0.002	0.57 (0.40–0.82)	0.002	0.43 (0.26–0.72)	0.001
Other dementia	2,302 (1.8)	1,366 (1.6)	0.89 (0.83–0.95)	<0.001	0.86 (0.81–0.92)	<0.001	0.90 (0.82–0.98)	0.016

Values are numbers (percentages) unless stated otherwise. HR, hazard ratio. Crude: without adjustment. Model 1: adjusted for age and gender. Model 2: included model 1 variables and additionally ethnicity (white, black, Asian, Chinese, mixed, or other ethnic group), education level, smoking status (never, former, or current), alcohol consumption (never, former, or current), hypertension (yes or no), antihypertensive drug use (yes or no), aspirin use (yes or no), lipid lowering medication (yes or no), other vitamin or mineral or dietary supplementation (yes or no), Townsend Deprivation Index, household income [<£18,000 (€21,489; $23,253), £18,000–£30,999, £ 31,000–£51,999, £52,000–£100,000, or >£100,000], physical activity (<150 or ≥150 min/week).

In the analyses, adjusted for age and gender (Model 1), fish oil users were significantly associated with lower risks of incident all-cause dementia, vascular dementia, frontotemporal dementia, and other dementia compared with fish oil non-users (HR = 0.88 [95% CI = 0.83–0.93], 0.78 [CI = 0.69–0.87], 0.57 [CI = 0.40–0.82], and 0.86 [CI = 0.81–0.92], respectively, all *P* < 0.01). In the multivariable adjusted models (Model 2), the adjusted HRs associated with fish oil supplementation for incident all-cause dementia, vascular dementia, frontotemporal dementia, and other dementia were 0.91 [CI = 0.84–0.97], 0.83 [CI = 0.71–0.97], 0.43 [CI = 0.26–0.72], and 0.90 [CI = 0.82–0.98]), respectively (all *P* < 0.05). However, there was no significant associations between fish oil supplementation and incident Alzheimer’s disease (HR = 1.00 [CI = 0.89–1.12], *P* = 0.977).

### Subgroup analyses

**FIGURE 1:**
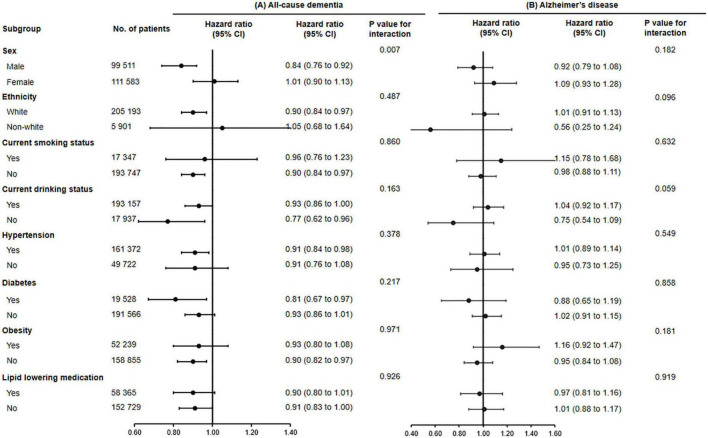
The association of fish oil supplementation and the risk of all-cause dementia **(A)** and Alzheimer’s disease **(B)** in different subgroups. Results were adjusted for age, gender, ethnicity (white, black, Asian, Chinese, mixed, or other ethnic group), education level, smoking status (never, former, or current), alcohol consumption (never, former, or current), hypertension (yes or no), antihypertensive drug use (yes or no), aspirin use (yes or no), lipid lowering medication (yes or no), other vitamin or mineral or dietary supplementation (yes or no), Townsend Deprivation Index, household income [<£18,000 (€21,489; $23,253), £18,000–£30,999, £31,000–£51,999, £52,000–£100,000, or >£100,000], physical activity (<150 or ≥150 min/week).

**FIGURE 2:**
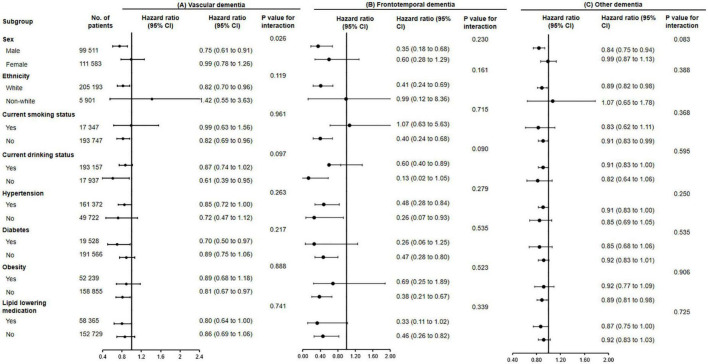
The association of fish oil supplementation and vascular dementia **(A)**, frontotemporal dementia **(B)**, and other dementia **(C)** in different subgroups. Results were adjusted for age, gender, ethnicity (white, black, Asian, Chinese, mixed, or other ethnic group), education level, smoking status (never, former, or current), alcohol consumption (never, former, or current), hypertension (yes or no), antihypertensive drug use (yes or no), aspirin use (yes or no), lipid lowering medication (yes or no), other vitamin or mineral or dietary supplementation (yes or no), Townsend Deprivation Index, household income [<£18,000 (€21,489; $23,253), £18,000–£30,999, £31,000–£51,999, £52,000–£100,000, or >£100,000], physical activity (<150 or ≥150 min/week).

Subgroup analyses were conducted according to potential risk factors that could affect the associations of fish oil supplementation with dementia and its subtypes (Figures 1, 2).

The associations between use of fish oil and the risk of all-cause dementia (*P* for interaction = 0.007) and vascular dementia were stronger among men (*P* for interaction = 0.026).

We conducted further stratified analyses according to the frequency of oily fish intake (Supplementary Table 1). Among individuals with oily fish intake <2 times/week, fish oil users were significantly associated with lower risks of incident all-cause dementia, vascular dementia, frontotemporal dementia and other dementia compared with fish oil non-users in the multivariable adjusted models (all *P* < 0.05). Likewise, there were no significant associations between fish oil supplementation and incident Alzheimer’s disease (*P* = 0.624). However, no significant associations between fish oil supplementation and dementia and its subtypes were found among those with oily fish intake ≥2 times/week (all *P* > 0.05).

## Discussion

In the large population-based cohort study of 211,094 participants, we provided the novel evidence that fish oil supplementation was associated with a significantly lower risks of incident dementia. Furthermore, fish oil supplementation was significantly associated with lower risks of incident vascular dementia, frontotemporal dementia, and other dementia; however, no significant association for incident Alzheimer’s disease was found. These associations were independent of risk factors, including age, gender, ethnicity, smoking and drinking status, hypertension, diabetes, obesity, use of medication, and any other supplements. Our findings indicate that habitual use of fish oils would be beneficial for the prevention of dementia in the general population, mainly for vascular dementia, frontotemporal dementia, and other dementia. These findings have clinical practice and public health implications for the improvement of cognition decline and the prevention of dementia.

Our findings indicated that fish oil supplements were associated with lower risk of incident all-cause dementia in older individuals. Several epidemiological studies reported that dietary fish oil intake was associated with lower risks of dementia (Huang et al., 2005; Nozaki et al., 2021) or lower cognitive decline (van Gelder et al., 2007; Thomas et al., 2020). In a meta-analysis from 21 cohort studies (181,580 participants) reported that dietary fish oil intake was associated with lower risks of cognitive impairment and incident dementia (Zhang et al., 2016). Furthermore, several small clinical trials indicated that omega-3 supplementation significantly slowed cognitive decline in at-risk elderly people or patients with prodromal Alzheimer’s disease (Arellanes et al., 2020; Soininen et al., 2021). Other larger clinical trials, however, have reported inconsistent and mixed results (Andreeva et al., 2011; Chew et al., 2015). For instance, one RCT of 2,911 patients with stable myocardial infarction reported no effect of n-3 fatty acids supplementation on cognitive decline (Geleijnse et al., 2012). However, these studies did not provide conclusive information needed to formulate evidence-based clinical guidelines for dementia management.

One possible explanation is that those studies could lack sufficient sample sizes or sufficient events. Thus, insufficient sample sizes in RCTs not only generated the limited statistical power to detect the clinical effect of fish oil supplementation, but also limited the ability to examinate the influence of the potential confounding factors (Li et al., 2020). Another possible explanation is that the lack of protection from omega-3 fatty acids reported in previous clinical trials could be due to the dose and period of supplementation. For example, in two trials of cognitively healthy adults, the duration of follow-up was less than 6 months (van de Rest et al., 2008; Arellanes et al., 2020); nevertheless, by prolonging interventional duration, the LipiDiDiet study showed significant cognitive benefits with 36-months supplementation (Soininen et al., 2021). Furthermore, some trials used low doses of omega-3 fatty acids (Geleijnse et al., 2012; Phillips et al., 2015). However, these studies did not provide information regarding on the dose and the best duration of fish oil supplements that needed to achieve a clinically meaningful effect.

Notably, these RCT evidences are more limited and less convincing, with cognition or brain volume as main study endpoints (Chiu et al., 2008; Quinn et al., 2010; Arellanes et al., 2020), and few primary preventions study with incident dementia as an outcome measure. The present study, to our knowledge, is the first study with incident dementia as primary outcome measure to investigate the association of fish oil supplements with dementia prevention in real life settings of large-scale cohort. Our results provided novel evidence that fish oil supplementation was inversely associated with the risk of incident all-cause dementia with 11.7 years follow-up. These findings support that fish oil supplements may be beneficial for dementia prevention in clinical practice.

In the present study, we found no significant associations of fish oil supplementation with incident Alzheimer’s disease. Consistently, several epidemiological studies also reported no significant associations between dietary omega-3 fatty acids intakes and long-term risk of Alzheimer’s disease (Devore et al., 2009; Kröger et al., 2009). In contrast, some observational studies found that higher consumption of fish or intake of dietary omega-3 fatty acids were associated with lower risks of Alzheimer’s disease (Huang et al., 2005; Barberger-Gateau et al., 2007). Nevertheless, several RCTs reported an overall negligible, or no benefits of omega-3 fatty acids supplements on cognitive function in patients with Alzheimer’s disease (Chiu et al., 2008; Quinn et al., 2010; Phillips et al., 2015). In LipiDiDiet study of 311 patients with prodromal Alzheimer’s disease, the multinutrient combination (contains DHA and EPA) also showed no significant effect on the neuropsychological test battery score primary endpoint during the 24-month intervention (Soininen et al., 2017). One meta-analysis of 22,402 participants also reported no significantly inverse association between dietary omega-3 fatty acids intake and the risk of Alzheimer’s disease (Wu et al., 2015). In addition, information regarding the associations of fish oil supplements with incident Alzheimer’s disease is still limited. Our data indicated that fish oil supplements might have no benefits for the prevention of Alzheimer’s disease. The possible explanation is that Alzheimer’s disease is highly heterogeneous and fish oil supplements may interact with apolipoprotein E (APOE) genotype and stage of Alzheimer’s disease pathologic changes (Gatz et al., 2006; Ridge et al., 2016; Arellanes et al., 2020; Scheltens et al., 2021). Therefore, our data did not support the use of omega-3 fatty acids supplements for the prevention of Alzheimer’s disease, which was consistent with the current dementia management guideline (Hort et al., 2010).

In addition, our findings demonstrated that fish oil supplementation were inversely associated with the lower risks of incident vascular dementia and frontotemporal dementia. To date, there is limited evidence regarding the effects of fish oil supplements for the prevention of vascular dementia and frontotemporal dementia in clinical practice. Our findings suggested that it is attractive to take fish oil into consideration for preventing vascular dementia and frontotemporal dementia as potential options. Several mechanisms could explain the benefits for vascular dementia and frontotemporal dementia prevention derived from fish oil supplementation. Firstly, numerous studies have indicated that supplementation with omega-3 fatty acids has beneficial effects on cardiometabolic health, such as blood pressure, plasma triglycerides and endothelial function (Preston Mason, 2019; Skulas-Ray et al., 2019), all of which would exert a protective effect against the development of vascular dementia and frontotemporal dementia. Secondly, omega-3 fatty acids have been proved to have antioxidant and anti-inflammatory properties that could be clinically beneficial (Bourre, 2006; Bright et al., 2019). Thirdly, studies have reported that fish oil can reduce thrombosis and promote neurogenesis (Marchioli, 1999; Youdim et al., 2000). Additionally, our findings demonstrated that fish oil supplementation produced a significant protective effect on the onset of frontotemporal dementia. Consistent with our findings, previous study reported that fish oil consumption may play a role in suppressing dystrophic neurites formation through the reduction of tau hyperphosphorylation (Jović et al., 2019), and thus reduce the risk of frontotemporal dementia. Further clinical trials need to determine the effects of fish oil supplements for the prevention of vascular dementia and frontotemporal dementia in clinical practice.

### Strengths and limitations

The major strength of the present study was its population-based cohort, which provided a large number of outcome events and adequate statistical power to investigate the effectiveness of fish oil supplementation on all-cause dementia and dementia subtypes in a real life setting over a long-term follow-up period. Furthermore, detailed information was available on the considerable medication, lifestyle habits, supplements or drug use, and demographic data, enabling us to minimize confounding factors through careful adjustment for a wide range of covariates.

There were several potential limitations in the present study. First, the study participants were simply stratified by fish oil uses or no-users according to an electronic questionnaire at baseline, lacking of detailed information of fish oil supplements such as formulation, dose and using duration. Therefore, it is difficult to evaluate dose-response associations between fish oil supplementation and incident dementia. Second, although a series of known potential confounders has been adjusted in our analyses, we cannot completely exclude the possibility of residual confounders in the present study. Third, it is difficult to distinguish the effects of a healthy lifestyle from the habitual use of fish oil supplements in determining risks of dementia in an observational study. Fourth, these results cannot be generalized to other ethnic populations since a considerable proportion (97.2%) of participants in UK Biobank cohort is white people. Fifth, the relatively low response rate (5.45% of 9,238,453 invited individuals) that consented to join the study cohort might have contributed to selection bias and underestimation of dementia incidence (Fry et al., 2017). Finally, misclassification of exposure remains a possibility while the overall accuracy of obtaining data is good, which is likely to have biased these findings (Lourida et al., 2019).

## Conclusion

Among older adults, regular fish oil supplementation was inversely associated with the risks of incident all-cause dementia and its subtypes, such as vascular dementia, frontotemporal dementia, and other dementia. However, no significant associations were found for Alzheimer’s disease. These findings support that habitual use of fish oils may be beneficial for the prevention of dementia in clinical practice. Future studies are needed to determine the appropriate dose and period of fish oil supplements that influences the ability to achieve a clinically meaningful effect on the prevention of dementia.

## Data availability statement

The datasets presented in this study can be found in online repositories. The names of the repository/repositories and accession number(s) can be found below: www.ukbiobank.ac.uk.

## Ethics statement

The UK Biobank received ethical approval from the Research Ethics Committee (REC reference for UK Biobank 11/NW/0382) and participants provided written informed consent.

## Author contributions

YH and HZ contributed to conception and design of the study. HZ supervised the study. JL, DG, LY, DL, BX, and CH organized the database. YH, PZ, and JL performed the statistical analysis. YH wrote the first draft of the manuscript. YH, YD, and PZ wrote sections of the manuscript. All authors contributed to the article and approved the submitted version.

## Footnotes

1
http://www.ukbiobank.ac.uk
