# Supplementary material for: Associations of fish oil supplementation with incident dementia: Evidence from the UK Biobank cohort study
Source: Front Neurosci. 2022 Sep 7;16:910977. doi: 10.3389/fnins.2022.910977 (PMC9489907; doi:10.3389/fnins.2022.910977)
Supplement: Supplementary file 1 [file Table_1.docx]

**Supplementary Table 1 | Associations of fish oil supplementation with the risk of dementia stratified by the frequency of oily fish intake.**

| **Outcomes** | **Fish oil non-users** | **Fish oil users** | **Crude** | | **Model 1** | | **Model 2** | |
| --- | --- | --- | --- | --- | --- | --- | --- | --- |
|  | **(n=127 811)** | **(n=83 283)** | **HR (95% CI)** | **P value** | **HR (95% CI)** | **P value** | **HR (95% CI)** | **P value** |
| **Oily fish intake *( n, %)*** |  |  |  |  |  |  |  |  |
| **<2 times/week (n=164 462）** | 102 015 (79.8) | 62 447 (75.0) |  |  |  |  |  |  |
| All cause dementia | 2 521 (2.5) | 1 346 (2.2) | 0.85 (0.80 to 0.91) | <0.001 | 0.83 (0.77 to 0.88) | <0.001 | 0.86 (0.79 to 0.94) | <0.001 |
| Alzheimer's disease | 1 036 (1.0) | 621 (1.0) | 0.96 (0.87 to 1.06) | 0.390 | 0.92 (0.83 to 1.01) | 0.093 | 0.97 (0.85 to 1.10) | 0.624 |
| Vascular dementia | 611 (0.6) | 284 (0.5) | 0.74 (0.64 to 0.85) | <0.001 | 0.72 (0.63 to 0.83) | <0.001 | 0.82 (0.68 to 0.98) | 0.033 |
| Frontotemporal dementia | 85 (0.1) | 27 (0.04) | 0.51 (0.33 to 0.78) | 0.002 | 0.52 (0.34 to 0.80) | 0.003 | 0.42(0.23 to 0.79) | 0.007 |
| Other dementia | 1774 (1.7) | 935 (1.5) | 0.84 (0.78 to 0.91) | <0.001 | 0.82 (0.75 to 0.88) | <0.001 | 0.85 (0.76 to 0.94) | 0.002 |
| **≥2 times/week (n=46 632)** | 25 796 (20.2) | 20 836 (25.0) |  |  |  |  |  |  |
| All cause dementia | 769 (3.0) | 638 (3.1) | 1.01 (0.91 to 0.13) | 0.816 | 0.99 (0.89 to 1.10) | 0.840 | 0.99 (0.87 to 1.14) | 0.910 |
| Alzheimer's disease | 315 (1.2) | 290 (1.4) | 1.12 (0.96 to 1.32) | 0.152 | 1.10 (0.94 to 1.29) | 0.258 | 1.07 (0.86 to 1.32) | 0.056 |
| Vascular dementia | 189 (0.7) | 140 (0.7) | 0.91 (0.73 to 1.13) | 0.369 | 0.88 (0.71 to 1.10) | 0.264 | 0.84 (0.63 to 1.12) | 0.231 |
| Frontotemporal dementia | 27 (0.1) | 15 (0.1) | 0.68 (0.36 to 1.28) | 0.234 | 0.68 (0.36 to 1.29) | 0.239 | 0.43 (0.18 to 1.02) | 0.056 |
| Other dementia | 528 (2.1) | 431 (2.1) | 1.00 (0.88 to 1.13) | 0.943 | 0.97 (0.86 to 1.11) | 0.680 | 1.02 (0.87 to 1.21) | 0.797 |

Values are numbers (percentages) unless stated otherwise. HR=hazard ratio.

Crude: without adjustment.

Model 1: adjusted for age and gender.

Model 2: included model 1 variables and additionally ethnicity (white people, black people, Asian people, Chinese, mixed, or other ethnic group), education level, smoking status (never, former, or current), alcohol consumption (never, former, or current), hypertension (yes or no), antihypertensive drug use (yes or no), aspirin use (yes or no), lipid lowering medication (yes or no), other vitamin or mineral or dietary supplementation (yes or no), Townsend Deprivation Index, household income (<£18 000 (€21 489; $23 253), £18 000-£30 999, £31 000-£51 999, £52 000-£100 000, or >£100 000), physical activity (<150 or ≥150 min/week).
