# Supplementary material for: Associations of fish oil supplementation with incident dementia: Evidence from the UK Biobank cohort study
Source: Front Neurosci. 2022 Sep 7;16:910977. doi: 10.3389/fnins.2022.910977 (PMC9489907; doi:10.3389/fnins.2022.910977)
Supplement: Supplementary file 2 [file Data_Sheet_1.doc]

**Supplementary File: Field IDs**

| **Field IDs** | **Description** |
| --- | --- |
| 10007 | Vitamin and mineral supplements (pilot) |
| 6179 | Mineral and other dietary supplements |
| 21022 | Age at recruitment |
| 21003 | Age when attended assessment centre |
| 53 | Date of attending assessment centre |
| 31 | Sex |
| 6138 | Qualifications |
| 10722 | Qualifications (pilot) |
| 21000 | Ethnic background |
| 21001 | Body mass index (BMI) |
| 1329 | Oily fish intake |
| 20116 | Smoking status |
| 20117 | Alcohol drinker status |
| 1558 | Alcohol intake frequency. |
| 2443 | Diabetes diagnosed by doctor |
| 4041 | Gestational diabetes only |
| 10844 | Gestational diabetes only (pilot) |
| 6150 | Vascular/heart problems diagnosed by doctor |
| 6177 | Medication for cholesterol, blood pressure or diabetes |
| 6153 | Medication for cholesterol, blood pressure, diabetes, or take exogenous hormones |
| 6154 | Medication for pain relief, constipation, heartburn |
| 10723 | Vitamin supplements (pilot) |
| 6155 | Vitamin and mineral supplements |
| 10854 | Other dietary supplements (pilot) |
| 4079 | Diastolic blood pressure, automated reading |
| 94 | Diastolic blood pressure, manual reading |
| 93 | Systolic blood pressure, manual reading |
| 4080 | Systolic blood pressure, automated reading |
| 30760 | HDL cholesterol |
| 30780 | LDL direct |
| 30870 | Triglycerides |
| 30690 | Cholesterol |
| 30740 | Glucose |
| 30750 | Glycated haemoglobin (HbA1c) |
| 41270 | Diagnoses - ICD10 |
| 41280 | Date of first in-patient diagnosis - ICD10 |
| 40000 | Date of death |
| 40001 | Underlying (primary) cause of death: ICD10 |
| 40002 | Contributory (secondary) causes of death: ICD10 |
| 20002 | Non-cancer illness code, self-reported |
| 10004 | Medication for pain relief, constipation, heartburn (pilot) |
| 131286 | Date I10 first reported (essential (primary) hypertension) |
| 131294 | Date I15 first reported (secondary hypertension) |
| 1339 | Non-oily fish intake |
| 189 | Townsend deprivation index at recruitment |
| 884 | Number of days/week of moderate physical activity 10+ minutes |
| 894 | Duration of moderate activity [Physical activity](https://biobank.ndph.ox.ac.uk/ukb/label.cgi?id=100054) |
| 904 | Number of days/week of vigorous physical activity 10+ minutes |
| 914 | Duration of vigorous activity |
| 738 | Average total household income before tax |
| 10877 | Average total household income before tax (pilot) |
